# Reply: Adjuvant chemoradiotherapy is no alternative to intensified neoadjuvant chemoradiotherapy for local and systemic control in patients with locally advanced rectal cancer

**DOI:** 10.1038/sj.bjc.6603339

**Published:** 2006-10-03

**Authors:** G Klautke, R Fietkau

**Affiliations:** 1 Department of Radiotherapy Sudring, University of Rostock, Sudring 75, Rostock 18059, Germany

**Sir,**

Dr Papagrigoriadis’ main points of criticism of our neoadjuvant strategy for treatment of rectal cancer are the toxicity of chemotherapy and subsequent surgery and the potential risk of overtreating the patient owing to overstaging. We respond as follows:

1. Neoadjuvant chemoradiotherapy has lower rates of acute toxicity and late toxicity than adjuvant chemoradiotherapy regimens of comparable intensity. This was clearly demonstrated by the German Rectal Cancer Study (Sauer *et al*, 2004). All of the studies cited by Dr Papagrigoriadis are trials investigating intensified neoadjuvant chemoradiotherapy. To our knowledge, no comparable studies or the corresponding data on the toxicity of intensified adjuvant chemoradiotherapy exist. In our opinion, postoperative intensive chemoradiotherapy is not feasible in light of evidence that an already investigated adjuvant treatment scheme calling for 5-fluorouracil (5-FU) (2.6 g m^−2^/24 h; weekly) and folinic acid concurrent with radiation ‘*should not be recommended for further use in postoperative adjuvant treatment*’ owing to toxicity reasons (Dencausse *et al*, 2001).
2. The main goals of intensive chemoradiotherapy are to achieve higher rates of local control with lower rates of distant metastasis. Studies by Mitchell's team and our own suggest that this is possible (Klautke *et al*, 2005; Mitchell *et al*, 2005). We hope that follow-up studies performed in the scope of the German Rectal Cancer Study and the PETAC 6 study will provide a definitive answer to this question.
3. We do not share Dr Papagrigoriadis’ opinion that patients who undergo resection after neoadjuvant chemoradiotherapy have a 3–5% higher risk of mortality. Neither the NSABP R-03 Study nor the German Rectal Cancer Study showed an overall increase in the rate of perioperative complications in neoadjuvant treatment arms compared with the adjuvant treatment arms. Furthermore, a number of phase I and II studies on intensive neoadjuvant chemoradiotherapy did not reveal any higher rates of perioperative complications owing to intensification. More than 100 patients have received intensive neoadjuvant chemoradiotherapy in four studies performed at our hospital (Klautke *et al*, 2006). None of these patients died owing to perioperative complications.
4. We conscientiously reported on complications associated with our intensive treatment regimen. Fatalities occurred in two of 28 patients. However, these deaths must be assessed by means of a differential analysis, which we included in the publication: One patient died of 5-FU-related toxicity (sudden cardiac death) – a rare and tragic complication of the drug (Tsavaris *et al*, 2002; Alter *et al*, 2006). This is not attributable to the treatment sequence and could also happen in an adjuvant treatment setting. Another patient developed a dose-limiting toxicity (CTC grade 4 diarrhoea) during the dose-finding stage with capecitabine and was then transferred to the intensive care unit for safety reasons. The patient fully recovered from her abdominal complaints and was supposed to be discharged before the planned resection surgery but she suddenly developed Klebsiella pneumonia and died. This death was associated with intensive radiochemotherapy (RCT) but could also occur in adjuvant RCT with 5-FU. It is important to report these experiences because the occurrence of the dose-limiting toxicity (grade 4 diarrhoea) means that the administered dose should no longer be used. The intensity was therefore decreased in the later studies, and no cases of severe diarrhoea developed. None of the other 100+ patients who received intensive neoadjuvant chemoradiotherapy at our hospital died of treatment complications. By adjusting the capecitabine dosage, with treatment on days 1–14 and 21–35 separated by a 1-week break in treatment, we successfully reduced the rate of diarrhoea as an acute toxicity to a level of roughly 10% (CTC grade 3 diarrhoea) without decreasing the efficacy of treatment (Klautke *et al*, 2006).
5. We agree with Dr Papagrigoriadis that overstaging is a problem associated with a risk of overtreatment. Overstaging occurred in approximately 20% of patients treated in the German Rectal Cancer Study. However, the lack of performance of a pretreatment pelvic MRI examination could be the reason from some cases of overstaging in this study. In addition, patients who had sonographically confirmed lymph node involvement without stage uT3 or uT4 rectal cancer were also included in the study. uT3/uT4 status was a prerequisite for inclusion in our studies. This diagnosis cannot be made based on ultrasound evidence of lymph node involvement alone. We are convinced that consistent use of endosonography and MRI with additional PET-CT as needed (even if difficult to perform in the pelvic region), when performed by an experienced examiner, can significantly reduce the risk of overstaging.

In summary, we conclude that intensive neoadjuvant chemoradiotherapy of UICC stage II and III rectal cancer can produce promising results with generally acceptable toxicity. The current evidence suggests that this treatment strategy may improve local control whereas reducing the rate of distant metastasis. However, intensive neoadjuvant chemoradiotherapy should be carried out within the framework of clinical trials designed to demonstrate the effects of this treatment strategy.
